# Nutrient and Total Polyphenol Contents of Dark Green Leafy Vegetables, and Estimation of Their Iron Bioaccessibility Using the In Vitro Digestion/Caco-2 Cell Model

**DOI:** 10.3390/foods6070054

**Published:** 2017-07-22

**Authors:** Francis Kweku Amagloh, Richard Atinpoore Atuna, Richard McBride, Edward Ewing Carey, Tatiana Christides

**Affiliations:** 1 Food Science & Technology Department, Faculty of Agriculture, University for Development Studies, Nyankpala, Ghana; 2 Department of Agricultural Biotechnology and Molecular Biology, Faculty of Agriculture, University for Development Studies, Nyankpala, Ghana; richtuna024@gmail.com; 3 Department of Life & Sports Science, Faculty of Engineering and Science, University of Greenwich at Medway, Central Avenue Chatham Maritime, Kent ME4 4TB, UK; richard_mcbride@hotmail.co.uk (R.M.); T.Christides@greenwich.ac.uk (T.C.); 4 International Potato Centre, Kumasi, Ghana; e.carey@cgiar.org

**Keywords:** β-carotene, Caco-2 cell, iron bioaccessibility, leafy vegetable, polyphenols

## Abstract

Dark green leafy vegetables (DGLVs) are considered as important sources of iron and vitamin A. However, iron concentration may not indicate bioaccessibility. The objectives of this study were to compare the nutrient content and iron bioaccessibility of five sweet potato cultivars, including three orange-fleshed types, with other commonly consumed DGLVs in Ghana: cocoyam, corchorus, baobab, kenaf and moringa, using the in vitro digestion/Caco-2 cell model. Moringa had the highest numbers of iron absorption enhancers on an “as-would-be-eaten” basis, β-carotene (14169 μg/100 g; *p* < 0.05) and ascorbic acid (46.30 mg/100 g; *p* < 0.001), and the best iron bioaccessibility (10.28 ng ferritin/mg protein). Baobab and an orange-fleshed sweet potato with purplish young leaves had a lower iron bioaccessibility (6.51 and 6.76 ng ferritin/mg protein, respectively) compared with that of moringa, although these three greens contained similar (*p* > 0.05) iron (averaging 4.18 mg/100 g) and β-carotene levels. The ascorbic acid concentration of 25.50 mg/100 g in the cooked baobab did not enhance the iron bioaccessibility. Baobab and the orange-fleshed sweet potato with purplish young leaves contained the highest levels of total polyphenols (1646.75 and 506.95 mg Gallic Acid Equivalents/100 g, respectively; *p* < 0.001). This suggests that iron bioaccessibility in greens cannot be inferred based on the mineral concentration. Based on the similarity of the iron bioaccessibility of the sweet potato leaves and cocoyam leaf (a widely-promoted “nutritious” DGLV in Ghana), the former greens have an added advantage of increasing the dietary intake of provitamin A.

## 1. Introduction

It is generally accepted that dark green leafy vegetables (DGLVs) are important sources of micronutrients such as iron and vitamin A. For example, on the basis of compositional data, DGLVs were reported to contribute about 19–39% of iron and 42–68% of vitamin A [1] in the diets of rural South Africans. However, iron and vitamin A deficiencies are perennial malnutrition problems in developing countries where DGLVs are important food ingredients [2,3]. One of the common food ingredients, possibly with a high concentration of micronutrients such as iron and β-carotene (provitamin A), are the greens. However, Cercamondi and co-workers [4] reported that sauce prepared from amaranth (*Amaranthus cruentus*) or Jew’s mallow/corchorus (*Corchorus olitorius*) and examples of DGLVs, eaten with a thick maize paste by young Burkinabe women, did not increase the amount of iron absorbed. An inadequate dietary intake of bioavailable iron and vitamin A could be the primary cause of iron and vitamin A deficiencies. Therefore, the bioaccessibility of minerals from food may not solely depend on their concentration, but also on other constituents in the food.

Polyphenols and phytates in cereal and leguminous foods have been shown to limit the bioaccessibility, and consequently, the bioavailability of essential micronutrients including iron, calcium and zinc [5,6]; these staples are usually consumed with DGLVs that may also contain significant levels of these inhibitors. In a human feeding trial conducted by Garcia-Casal and co-workers [7], it was found that β-carotene enhances iron absorption when added to cereal-based diets. This finding was confirmed using Caco-2 cells as a model for iron availability [8]. Thus, the consumption of these greens, reported to be rich in micronutrients such as β-carotene [9,10], should have a double impact as a provitamin A dietary source, and also as an enhancer of iron absorption. However, this was contrary to the findings of Cercamondi and co-workers [4]. This calls for the need to investigate the iron bioaccessibility of commonly consumed DGLVs in Ghana, as anaemia (not categorised) prevalence has consistently been stated to be above 73% for children under 5 years, and at 35% among women of reproductive age in northern Ghana [11,12,13], where the consumption of greens is high. Vitamin A deficiency among Ghanaian children under 5 years was approximately 79% [14], expectedly, as micronutrient deficiencies usually occur together. In Ghana, DGLVs have been reported to be reliable sources of β-carotene for the majority of the population [10].

Amaranth and jute are widely consumed DGLV in northern Ghana, in addition to others such as baobab (*Adansonia digitata*), and moringa (*Moringa oleifera)* [15]. Sweet potato (*Ipomoea batatas*) is available in northern Ghana [16], but is mainly cultivated for the roots. Sweet potato leaf has been reported to contain appreciable levels of vitamin A, iron and other essential nutrients, including water-soluble vitamins [17,18], and the crop can be cultivated with low agricultural inputs [19]. Also, it has been reported that the sweet potato leaves have higher caffeoylquinic acid derivatives (polyphenols) than commercial vegetables with physiological functions, due to their enhanced antimutagenic and antioxidative properties [20]. Although the polyphenols have health benefits, they may compromise the iron bioaccessibility from the DGLVs. Different polyphenols exist, and have differing effects on the iron bioaccessibility [21,22,23,24]. Based on the nutrient superiority of the sweet potato leaf [17], it could serve as an alternative source of leafy vegetables to the populace in tropical regions of the world, particularly in Africa, where vitamin A and iron deficiencies often co-exist and remain public health problems [2,3]. The compositional data suggest that sweet potato and moringa leaves might be better sources of bioavailable iron, compared with other leafy green vegetables, as both have high levels of iron and β-carotene—a dietary factor that has been reported to improve iron bioaccessibility. However, the use of the greens of sweet potato as a leafy vegetable in Ghana is limited.

There is a need to do a comparative study of leaves commonly consumed, and sweet potato leaf before the latter could be suggested as an alternative green in Ghana, as a source of bioavailable iron or β-carotene. The in vitro digestion/Caco-2 cell model has been suggested to be less expensive than human trials [25,26], a more physiological tool for screening iron availability in comparison with solubility and dialysability methods, and an effective approach for predicting the iron bioaccessibility from food for humans [27,28]. Therefore, the in vitro digestion/Caco-2 cell model, with ferritin formation as a marker for iron absorption, was used to measure the iron bioaccessibility of selected greens available in Ghana, in comparison with sweet potato leaves.

The objectives of this study were to compare the nutrient contents and iron bioaccessibility using the in vitro digestion/Caco-2 cell model of five different cultivars of sweet potato, with five other commonly consumed DGLVs in Ghana: cocoyam (*Xanthosoma sagittifolium*), corchorus, baobab, kenaf (*Hibiscus cannabinus*) and moringa.

## 2. Materials and Methods

### 2.1. Sample Cultivation and Collection

**Figure 1:**
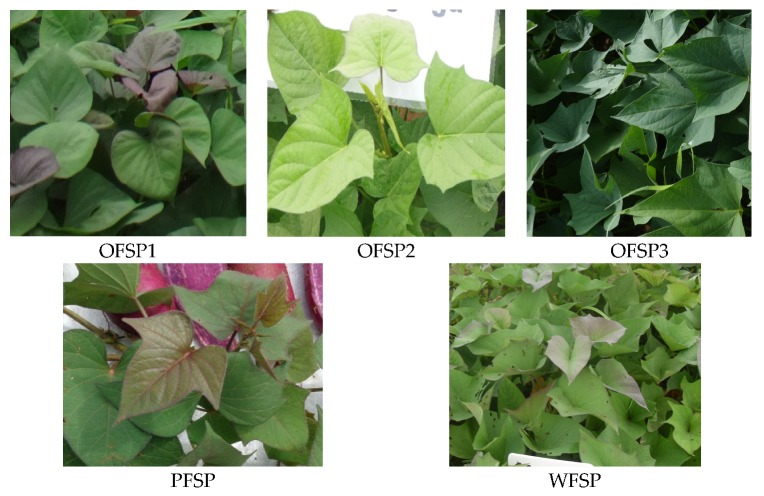
Cultivars of sweet potato (*Ipomoea batatas*) leaves used in the study. OFSP: orange-fleshed sweet potato; PFSP: purple-fleshed sweet potato; WFSP: white-fleshed sweet potato.

**Figure 2:**
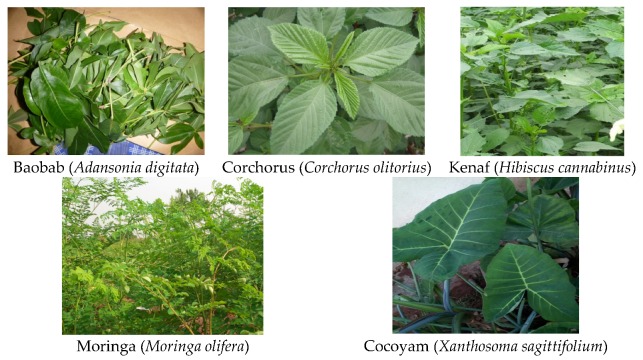
Commonly consumed dark green leafy vegetables (DGLVs) used in the study.

Five cultivars of sweet potato—three orange-fleshed (Coded OFSP1, OFSP2 and OFSP3), one purple-fleshed (PFSP), and one white-fleshed (WFSP)—and three other DGLVs, namely moringa, corchorus and kenaf (Figure 1 and Figure 2), were nursed in a screen house up to maturity (8 weeks). Each DGLV was cultivated in three replicates, and each replicate contained five pots of the particular green. Baobab and cocoyam were purposively sampled from three different geographical locations. Baobab leaves were collected from trees near settlements from the Upper East, Upper West and North regions, while cocoyam leaves were harvested from farmlands from the Ashanti, East and Brong-Ahafo regions of Ghana. The baobab was not nursed due to a relatively long time for the initiation of vegetative growth. Cocoyam is normally cultivated in the rainforest regions in Ghana and not in northern Ghana.

### 2.2. Sample Preparation

The replicates of the DGLVs were separately washed twice under running tap water and rinsed in distilled water; about two handfuls of DGLVs put into a stainless steel cup with 100 mL of distilled water added were covered with aluminium foil and boiled until soft, for between approximately 15 and 20 min. The cooked DGLVs were allowed to cool, and all the contents of the cup were transferred into coded, transparent, low-density polyethylene zip-lock bags, and stored in a freezer at −18 °C for 2 weeks. Prior to storage in the freezer, about 5 g aliquot portions were taken for moisture determination. The frozen samples were then freeze-dried (TK-118 Vacuum Freeze-Dryer, True Ten Industrial Company Limited, Taichung, Taiwan) for 72 h. The samples were then milled (Thomas Scientific, Dayton Electric Manufacturing Company Limited, Niles, IL, USA) and sieved into fine powder using a 60 mm sieve.

Triplicate aliquots of three-letter-coded powdered samples were couriered to the University of Greenwich at Medway, Chatham-Maritime United Kingdom, and Massey University, Palmerston North, New Zealand, from Ghana. The moisture determination of fresh leaves was performed in Ghana.

### 2.3. Compositional Analysis

#### 2.3.1. Moisture and Protein

The moisture contents of freshly harvested leaves and cooked leaves were gravimetrically determined using the forced air oven method (AOAC 925.10). For the milled freeze-dried samples, the vacuum oven protocol (AOAC 926.12), as published in the official methods of analysis of AOAC International [29], was used for the moisture determination.

The concentration of nitrogen in the freeze-dried greens was performed by the Dumas method (AOAC 968.06), and a nitrogen-to-protein conversion factor of 6.25 was used to quantify the amount of protein in the leaves on a fee-for-service basis by Massey University Nutrition Laboratory, Palmerston North, New Zealand.

#### 2.3.2. Mineral Analysis: Calcium, Iron, and Zinc

Approximately 0.50 g of the freeze-dried DGLV samples was microwave-digested using an accelerated reaction system (CEM MARS 5H with XP-1500 vessels) for 20 min at 400 psi and 1200 W. Subsequently, calcium, iron and zinc were quantified using an Inductively Coupled Plasma-Optical Emission Spectrometer (ICP-OES, Perkin–Elmer Optima 4300 DV, Perkin–Elmer, Coventry, UK) using protocols as previously described [30]. A certified reference material (ERMCD281, Sigma-Aldrich, UK) was included and run in parallel with the DGLV samples. The data obtained for all three minerals in the reference material were within 5% of the expected values.

#### 2.3.3. β-Carotene

Other researchers have described the extraction and quantification methods used in this study [31]. Averagely, 0.50 g of the freeze-dried samples of the leaves was used for the extraction. A certified reference material (BCR—485, Sigma-Aldrich now Merck, provided to Sigma-Aldrich from the European Commission Joint Research Centre, Institute for Certified Reference Materials and Measurements, Geel, Belgium) was included in three out of the five batches of extraction carried out on DGLV samples. A mean recovery of 128% was obtained for the β-carotene level for the reference material. Therefore, the values obtained for DGLVs were adjusted for a systematic error of 28%.

#### 2.3.4. Ascorbic Acid

The method for vitamin C determination as published by Lee and Coates [32] was carried out by the Massey University Nutrition Laboratory, Palmerston North, New Zealand, on a fee-for-service basis.

#### 2.3.5. Polyphenols

The Folin–Ciocalteu method described by Isabelle and co-workers [33] was used to quantify the total polyphenols in the samples, as gallic acid equivalents. The Nutrition Laboratory, Massey University, New Zealand Palmerston North, New Zealand, carried out the analysis on a fee-for-service basis.

### 2.4. In Vitro Digestion/Caco-2 Cell Model for Iron Availability

The iron availability from the freeze-dried DGLVs as received from Ghana was assessed using the TC7 Caco-2 cell clone (INSERM U505, Paris, France) from cell passages 42–45 in the in vitro digestion/Caco-2 cell model, as previously described [34], with slight modification. Averagely, 0.5 g rather than 1 g of the sample was weighed for the assessment, as 1 g of the starting material led to a matrix that was too viscous for the multiple mixing and pH adjustments required in this method. Cells were grown in six-well tissue culture plates for the experiments and maintained in DMEM supplemented with 10% *v*/*v* foetal bovine serum (FBS). On days 12 and 13, cell media were changed to MEM without FBS, as in the method developed by Glahn [35,36], to ensure low iron media, but optimal expression of Caco-2 cell iron transport proteins [37]. On day 14, foods were subjected to in vitro digestion with a sequential addition of digestive enzymes to mimic exposure to the stomach and small intestine (pepsin at pH 2, followed by bile/pancreatin at pH 7). Digested foods (digestates) and controls, including a blank “No food/added iron” digestate, were then applied to Caco-2 cells through an upper chamber suspended over the plate wells, created using a 15 kD dialysis membrane fitted over a Transwell insert and held in place with a silicon ring. The membrane protected the cells from the digestive enzymes, and also mimicked the gut mucous layer by only allowing soluble iron of a selected size to be available for enterocyte absorption. Cells were treated for 2 h, the digestates were removed, and the cells were returned to the incubator. The cells were harvested for ferritin 24 h after the initiation of the digestive process. Ferritin was measured using a commercial enzyme-linked immunosorbent assay (Spectro ferritin, RAMCO Laboratories Inc., Stafford, TX, USA), and corrected for differing numbers of cell per tissue culture well by measurement of cell protein as an indicator of cell numbers; the cell protein was measured using the Pierce protein bicinchoninic acid assay. Ferritin values were expressed as ng ferritin/mg cell protein.

### 2.5. Statistical Analysis

The compositional data were converted to an “as-would-be-eaten” basis prior to statistical analysis, using the dry matter content obtained for the cooked samples prior to storage in the freezer. The univariate analysis, followed by Tukey’s studentised range test with the significance set at *p* < 0.05, was used for the compositional data. For the in vitro digestion/Caco-2 cell model for iron availability, the data generated were normalised prior to using the general linear model procedure for one-factor analysis, and the results were presented as interval plots of the means with 95% confidence intervals. The Minitab 16.2.2 (Minitab Inc., State College, PA, USA) statistical package was employed for the data analysis.

## 3. Results

### 3.1. Compositional Profile

The data in Table 1 is expressed on the as-would-be-eaten basis, with the exception of the moisture value of the freshly harvested leaves. The moisture content of the sweet potato cultivars ranged from 83 to 87 g/100 g, and it was similar to other cultivars cultivated in China [38]. The greens of the sweet potato cultivars were generally not significantly different (*p* > 0.05) from each other for all the components analysed, with the exception of the total polyphenols.

OFSP1, Apomuden, a variety being promoted in Ghana because of the β-carotene content in the storage root [39], had approximately 1.7 times more total polyphenols than the other sweet potato cultivars. The leaves of the sweet potato cultivars were not distinctively superior in the levels of the micronutrients analysed, compared with the other DGLVs. However, OFSP1 contained appreciably higher levels of β-carotene (10,533 μg/100 g) and total polyphenols than the other greens, apart from the β-carotene level in moringa (1.3 times more), and the total polyphenols in baobab, which was about thrice higher. Although the roots of the OFSP cultivars are promoted as a dietary source of vitamin A, moringa leaves actually had the highest β-carotene concentration among the DGLVs investigated. Although the WFSP root is devoid of β-carotene [40], the amount of provitamin A in the leaf was more than that in the greens of OFSP2 and OFSP3.

In contrast, among the commonly consumed DGLVs, only baobab leaves contained the highest amount of calcium (*p* < 0.001): on average, about four times more. There was no significant difference in the iron concentration (*p* > 0.05), but the data showed that the iron level in baobab and moringa (4.59 ± 1.28 and 4.55 ± 1.88 mg/100 g, respectively) was higher. Previous data indicated that moringa contained 28.29 ± 0.05 mg/100 g of compositional iron [17], the highest compared with the seven sweet potato varieties in Ghana; the data in this study followed a similar trend.

Three of the DGLVs with notable amounts of ascorbic acid were moringa, baobab, and kenaf. The total polyphenols in baobab was the highest (1646.75 ± 69.44 mg GAE; *p* < 0.001) among all the DGLVs, including the sweet potato cultivars considered in this study. Moringa had a moderate content of total polyphenols, about one-fifth of that in Baobab (*p* < 0.05).

The concentration of zinc in the cocoyam leaf was 1.49 mg/100 g, about thrice more than the average of all the other DGLVs (*p* < 0.001). A similar trend of the zinc data between moringa and the sweet potato cultivars in this study was observed in a previous study in Ghana [17].

**Figure 3:**
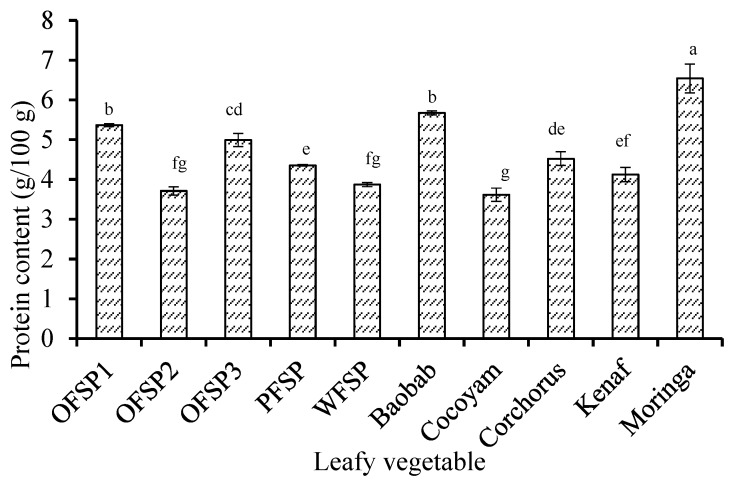
Protein content in “as-would-be-eaten” leafy vegetables. Bar values are means ± standard deviation (*n* = 3); bars with different letters ^(a–g)^ are significantly different (*p* < 0.0001). OFSP—orange-fleshed sweet potato (1, 2 and 3); PFSP—purple-fleshed sweet potato; and WFSP—white-fleshed sweetpotato.

Figure 3 shows the crude protein content of all the DGLVs, ranging from 3.62–6.54 g/100 g on the as-would-be-eaten basis. Moringa contained the highest protein (6.54 ± 0.36 g/100 g), and was significantly different (*p* < 0.05) from the next DGLV, baobab (5.67 ± 0.05 g/100 g), which was followed by two cultivars of sweet potato: OFSP1 (5.37 ± 0.04 g/100 g) and OFSP3 (4.99 ± 0.17 g/100 g). The two DGLVs with the lowest protein levels were WFSP (3.87 ± 0.05 g/100 g) and Cocoyam (3.62 ± 0.17 g/100 g). A trend between the protein data for moringa and the sweet potato cultivars was similar to a previous study in Ghana [17].

### 3.2. In Vitro Iron Bioaccessibility Using Caco-2 Cells as a Model

**Figure 4:**
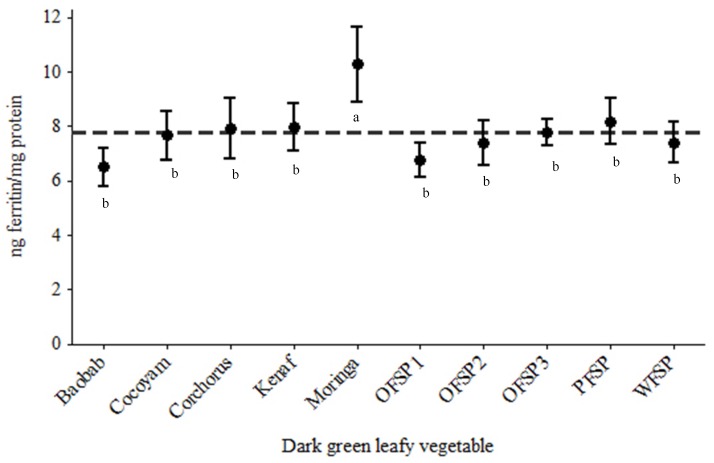
Ferritin formation per half a gram of freeze-dried green leafy vegetables. Vertical lines are means with 95% confidence intervals of ng ferritin/mg protein from the various greens (*n* = 12 for corchorus; *n* = 18 for OFSP1, PFSP, baobab, kenaf and moringa; and *n* = 21 for OFSP2, OFSP3, WFSP and cocoyam) normalised to the blank digest ferritin level; horizontal line indicates the overall mean of ng ferritin/mg protein; means with 95% confidence intervals with a different letter ^(a,b)^ are significantly different (*p* < 0.0001).

The data representing the in vitro iron bioaccessibility are shown in Figure 4. The overall mean of the iron bioaccessibility was 7.71 ng ferritin/mg protein. Moringa markedly had the best iron bioaccessibility, 10.28 ± 2.73 ng ferritin/mg protein, and was significantly different (*p* < 0.0001) from all the DGLVs investigated. 

The two greens (baobab and OFSP1) that could be ranked first and second in terms of the concentrations of total polyphenols had the lowest iron bioaccessibility using the Caco-2 cell model; their bioaccessibility was below the group mean. Conversely, cocoyam had an iron bioaccessibility at the overall mean, although it contained the lowest concentration of polyphenols. Apart from baobab, moringa and OFSP1, all the other DGLVs had a bioaccessibility similar to that of the overall mean.

To test the relationship between the iron bioaccesibility and some of the components (in the DGLVs investigated), a multiple linear regression was conducted (Table 2). Although the model explained about 75% of the variation in the iron bioaccesibility, it was the protein and iron levels that showed a marginal but positive effect such that a unit increase could respectively lead to 0.29 and 0.13 ng ferritin/mg protein formations in Caco-2 cells. However, with regard to zinc, an increase in its concentration resulted in a reduction of the ferritin formation by a 0.87 ng ferritin/mg protein. In this study, ascorbic acid and β-carotene (known enhancers of iron absorption), as well as total polyphenols (inhibitors of iron), had almost no effect on the iron bioaccessibility using the in vitro digestion/Caco-2 cell model.

## 4. Discussion

OFSP1 was the only sweet potato cultivar with purplish young leaves [41], among the five sweet potato genotypes evaluated in this study. This may have accounted for the highest total polyphenol content of OFSP1, compared to the other sweet potato cultivars. The difference in the iron data for moringa in this study compared to previous work [17] was due to how the data were reported. In the previous study, the result was reported on powdered samples, while in our study, it was on an as-would-be-eaten basis. Nonetheless, the trend of iron levels being the highest in moringa was also confirmed in this study. 

Although cocoyam leaf is widely consumed, and promoted in Ghana as a “nutritious” green to improve iron status (anecdotally), on the basis of its composition data, it was highest only in zinc, and lowest in β-carotene and total polyphenols, compared with the OFSP cultivars. Because both the sweet potato leaves and cocoyam had a similar iron bioaccessibility, the sweet potato leaves could be used in culinary preparations, and had an added advantage of increasing the dietary intake of β-carotene, compared to those of cocoyam.

Generally, the level of iron bioaccessibility from the DGLVs was relatively low (6–10 ng ferritin/mg protein) compared with our previous work on complementary food from the same laboratory (12–34 ng ferritin/mg protein) [34]. However, a strong comparison cannot be made between the data from the two studies, as different sample weights were used: 1 g in the previous work, and 0.5 g in the present study. A previous community-based feeding trial using Weanimix, which had an iron bioaccessibility of 17.32 ± 2.84 ng ferritin/mg protein [34], resulted in a poor iron status among older infants in Ghana [42,43]. The lower availability of iron in the greens in this study lends support to the finding of the work on young Burkinabe women, which resulted in no increase in iron absorption after eating Jew’s mallow with a thick maize paste [4].

As mentioned earlier, moringa contained the highest number of enhancers of iron absorption: β-carotene [7,8] and ascorbic acid [44]; although in this study their effect were not realised except for the concentration of iron. Additionally, the concentration of total polyphenols in this DGLV was moderate. The composition of nutrients in moringa, compared with the other DGLVs, may have contributed to it having the highest bioaccessibility of iron, as obtained from the in vitro Caco-2 cells model study. Although OFSP1 had significantly similar levels of β-carotene and iron to moringa, and one-third of the total polyphenols of baobab, its iron bioaccessibility was lower than for moringa, indicating that the reported caffeoylquinic acid derivatives in sweet potato leaves [20] may have limited the bioaccessibility of iron. Baobab had the lowest iron bioaccessibility, in spite of being one of the greens that contained the highest amounts of iron and ascorbic acid. This may have been attributed to the high concentration of total polyphenols [20], and not calcium, which is known to inhibit iron absorption [25,45]; and relative to the other DGLVs, suggesting that the polyphenols in baobab may be very inhibitory, even in the presence of endogenous ascorbic acid. However, the amount of calcium in the greens explicitly did not suggest inhibitory effects on iron, as moringa contained the second highest level of this mineral among all the DGLVs investigated, but had a markedly better iron availability. Therefore, predicting iron bioaccessibility based only on compositional data could lead to false conclusions.

The effect of the concentration of plant protein on the iron bioaccessibility cannot be explicitly substantiated in this study. Moringa, having the highest as-would-be-eaten protein, was the green with the highest bioaccessibility. Both baobab and OFSP1, which contained relatively high concentrations of protein compared to the rest of the DGLVs with the exception of Moringa, were those that recorded the lowest bioaccessibility of iron, although not significantly. Thus, from the data in this study, it is difficult to use the protein concentration to predict the iron bioaccessibility, although the regression analysis showed a direct effect. The inverse association between the zinc concentration and the index of iron bioaccessibility could be attributed to the cocoyam leaf, which had the highest zinc concentration and the lowest ferritin formation in the Caco-2 cells.

The major limitations of this study were that phytate and the constituents of the different classes of polyphenols were not quantified. The assay method previously used for phytate determination [46,47] gave very inconsistent results within replicates in this study; possibly the colour of DGLVs interfered with the spectrophotometer readings.

## 5. Conclusions

The studied greens varied in terms of calcium, iron and zinc levels. In addition, moringa had the highest levels of β-carotene and ascorbic acid. Baobab had the highest levels of calcium and total polyphenols. Within the limits of this study, iron bioaccessibility is influenced by a complex interplay of several components in DGLVs, including protein, ascorbic acid, β-carotene and total polyphenols. Moringa had the best iron bioaccessibility, and the lowest was found in baobab and one of the orange-fleshed sweet potatoes with purplish young leaves. Estimating iron bioaccessibility in greens based on the mineral concentration may lead to incorrect conclusions. Based on the similarity of the iron bioaccessibility of the sweet potato leaves and cocoyam leaf, the widely promoted “nutritious” DGLVs in Ghana, the former greens have an added advantage of increasing the dietary intake of provitamin A.

## Figures and Tables

**Table 1:** Moisture, micronutrient and total polyphenol levels per 100 g in DGLVs on an “as-would-be-eaten” basis ^#^.

DGLV	Moisture (g) ^¥^	Calcium (mg)	Iron (mg)	Zinc (mg)	β-Carotene (μg)	Ascorbic Acid (mg)	Total Polyphenols (mg GAE) ^†^
OFSP1	84.09 ± 0.34 ^c,d^	95.61 ± 8.01 ^c,d^	3.41 ± 0.36 ^a,b^	0.44 ± 0.01 ^b^	10,533 ^a,b^	0.74 ± 0.16 ^c^	506.93 ± 86.76 ^b^
OFSP2	84.76 ± 0.75 ^b,c,d^	81.04 ± 3.24 ^d^	1.89 ± 0.29 ^b^	0.42 ± 0.02 ^b^	8280 ^a,b,c^	0.50 ± 0.15 ^c^	356.69 ± 79.60 ^c^
OFSP3	87.24 ± 0.13 ^a^	103.25 ± 2.59 ^c,d^	2.58 ± 0.21 ^a,b^	0.36 ± 0.03 ^b^	7053 ^b,c^	0.45 ± 0.07 ^c^	336.38 ± 63.15 ^c,d,e^
PFSP	84.30 ± 0.26 ^c,d^	84.75 ± 8.83 ^c,d^	2.04 ± 0.36 ^b^	0.44 ± 0.04 ^b^	4472 ^b,c^	0.48 ± 0.03 ^c^	231.44 ± 49.77 ^c,d,e^
WFSP	83.91 ± 0.26 ^d^	87.02 ± 6.80 ^c,d^	3.27 ± 0.34 ^a,b^	0.40 ± 0.03 ^b^	9501 ^a,b,c^	0.34 ± 0.10 ^c^	234.86 ± 0.16 ^c,d,e^
Baobab	85.97 ± 0.53 ^b^	535.63 ± 22.93 ^a^	4.59 ± 1.28 ^a^	0.65 ± 0.03 ^b^	7166 ^b,c^	25.50 ± 0.01 ^b^	1646.75 ± 69.44 ^a^
Cocoyam	85.23 ± 0.64 ^b,c^	166.39 ± 15.13 ^b^	2.64 ± 0.16 ^a,b^	1.49 ± 0.47 ^a^	3911 ^c^	1.14 ± 0.01 ^c^	196.05 ± 10.96 ^e^
Corchorus	78.99 ± 0.38 ^f^	121.41 ± 3.61 ^c^	2.48 ± 0.23 ^a,b^	0.45 ± 0.02 ^b^	9298 ^a,b,c^	3.53 ± 0.58 ^c^	337.94 ± 16.44 ^c,d,e^
Kenaf	80.68 ± 0.18 ^e^	90.24 ± 17.76 ^c,d^	2.94 ± 0.25 ^a,b^	0.35 ± 0.05 ^b^	8959 ^a,b,c^	21.79 ± 1.54 ^b^	202.42 ± 9.29 ^d,e^
Moringa	78.81 ± 0.42 ^f^	186.22 ± 23.81 ^b^	4.55 ± 1.88 ^a^	0.77 ± 0.06 ^b^	14,169 ^a^	46.30 ± 4.78 ^a^	347.38 ± 14.59 ^c,d^
*p*-Value	<0.001	<0.001	0.002	<0.001	<0.001	<0.001	<0.001

^#^ Values are means ± standard deviation (*n* = 3), except for the β-carotene value (mean only); values with different letters ^(a–f)^ are significantly different (*p* < 0.0001); DGLV—dark green leafy vegetable; OFSP—orange-fleshed sweet potato; PFSP—purple-fleshed sweet potato; and WFSP—white-fleshed sweet potato. ^¥^ Moisture determined on freshly harvested leaves. ^†^ GAE—gallic acid equivalents.

**Table 2:** Effect of selected components (on an “as-would-be-eaten” basis) in DGLVs on iron bioaccesibility.

Variable ^#^	Estimate (Standard Error)	*p*-Value
Intercept	10.26 (3.24)	0.09
Calcium (mg/100 g)	−0.00 (0.03)	0.99
Iron (mg/100 g)	0.13 (1.18)	0.92
Zinc (mg/100 g)	−0.87 (1.81)	0.68
β-carotene (μg/100 g)	−0.00 (0.00)	0.35
Ascorbic acid (mg/100 g)	0.01 (0.08)	0.93
Total polyphenols (mg GAE/100 g)	−0.00 (0.01)	0.91
Protein (g/100 g)	0.29 (0.78)	0.74

^#^ Coefficient of determination (*R*^2^ = 74.62).

## References

[1] Faber M., Van Jaarsveld P., Laubscher R. (2007). The contribution of dark-green leafy vegetables to total micronutrient intake of two-to five-year-old children in a rural setting. Water SA.

[2] De Benoist B., McLean E., Egli I., Cogswell M. (2008). Worldwide Prevalence of Anaemia 1993–2005: WHO Global Database on Anaemia.

[3] World Health Organization (2009). Global Prevalence of Vitamin A Deficiency in Populations at Risk 1995–2005: WHO Global Database on Vitamin A Deficiency.

[4] Cercamondi C.I., Icard-Vernière C., Egli I.M., Vernay M., Hama F., Brouwer I.D., Zeder C., Berger J., Hurrell R.F., Mouquet-Rivier C. (2014). A higher proportion of iron-rich leafy vegetables in a typical Burkinabe maize meal does not increase the amount of iron absorbed in young women. J. Nutr..

[5] Davies N.T., Reid H. (1979). An evaluation of the phytate, zinc, copper, iron and manganese contents of, and Zn availability from, soya-based textured-vegetable-protein meat-substitutes or meat-extenders. Br. J. Nutr..

[6] Gautam S., Platel K., Srinivasan K. (2011). Promoting influence of combinations of amchur, β-carotene-rich vegetables and Allium spices on the bioaccessibility of zinc and iron from food grains. Int. J. Food Sci. Nutr..

[7] Garcia-Casal M.N., Layrisse M., Solano L., Baron M.A., Arguello F., Llovera D., Ramirez J., Leets I., Tropper E. (1998). Vitamin A and beta-carotene can improve nonheme iron absorption from rice, wheat and corn by humans. J. Nutr..

[8] Garcia-Casal M.N., Leets I. (2014). Carotenoids, but not vitamin A, improve iron uptake and ferritin synthesis by Caco-2 cells from ferrous fumarate and NaFe-EDTA. J. Food Sci..

[9] Van Jaarsveld P., Faber M., van Heerden I., Wenhold F., van Rensburg W.J., van Averbeke W. (2014). Nutrient content of eight African leafy vegetables and their potential contribution to dietary reference intakes. J. Food Compos. Anal..

[10] Takyi E.E.K. (1999). Children’s consumption of dark green, leafy vegetables with added fat enhances serum retinol. J. Nutr..

[11] ORC Macro Ghana Demographic and Health Survey 2003. http://www.dhsprogram.com/pubs/pdf/FR152/FR152.pdf.

[12] Ghana Statistical Service (GSS), Ghana Health Service (GHS), ICF International Ghana Demographic and Health Survey 2014. https://dhsprogram.com/pubs/pdf/FR307/FR307.pdf.

[13] Ghana Statistical Service (GSS), Ghana Health Service (GHS), ICF Macro Ghana Demographic and Health Survey 2008. http://www.dhsprogram.com/pubs/pdf/FR221/FR221[13Aug2012].pdf.

[14] World Health Organization WHO Global Database on Vitamin A Deficiency. http://www.who.int/vmnis/vitamina/data/database/countries/gha_vita.pdf.

[15] Amagloh F.K., Nyarko E.S. (2012). Mineral nutrient content of commonly consumed leafy vegetables in northern Ghana. Afr. J. Food Agric. Nutr. Dev..

[16] Dittoh S. (2004). Improving availability of nutritionally adequate and affordable food supplies at community levels in West Africa. International Workshop: Food-Based Approaches for a Healthy Nutrition in West Africa, Proceedings of the 2nd International Workshop, Ouagadougou, Burkina Faso, 23–28 November 2003.

[17] Oduro I., Ellis W., Owusu D. (2008). Nutritional potential of two leafy vegetables: *Moringa oleifera* and *Ipomoea batatas* leaves. Sci. Res. Essays.

[18] Barrera W.A., Picha D.H. (2014). Ascorbic acid, thiamin, riboflavin, and vitamin B6 contents vary between sweetpotato tissue types. HortScience.

[19] Faber M., Laurie S.M., van Jaarsveld P.J. (2013). Total β-carotene content of orange sweetpotato cultivated under optimal conditions and at a rural village. Afr. J. Biotechnol..

[20] Islam S. Antimutagenicity and antioxidant activity in the Ipomoea batatas L. genotypes in relation to polyphenolics. Proceedings of the International Conference on Advances in Agricultural, Biological & Environmental Sciences.

[21] Tako E., Beebe S., Reed S., Hart J., Glahn R. (2014). Polyphenolic compounds appear to limit the nutritional benefit of biofortified higher iron black bean (*Phaseolus vulgaris* L.). Nutr. J..

[22] Tako E., Reed S.M., Budiman J., Hart J.J., Glahn R.P. (2015). Higher iron pearl millet (*Pennisetum glaucum* L.) provides more absorbable iron that is limited by increased polyphenolic content. Nutr. J..

[23] Petry N., Egli I., Campion B., Nielsen E., Hurrell R. (2013). Genetic reduction of phytate in common bean (*Phaseolus vulgaris* L.) seeds increases iron absorption in young women. J. Nutr..

[24] Abizari A.-R., Moretti D., Schuth S., Zimmermann M.B., Armar-Klemesu M., Brouwer I.D. (2012). Phytic acid-to-iron molar ratio rather than polyphenol concentration determines iron bioavailability in whole-cowpea meal among young women. J. Nutr..

[25] Glahn R.P., Rassier M., Goldman M.I., Lee O.A., Cha J. (2000). A comparison of iron availability from commercial iron preparations using an in vitro digestion/Caco-2 cell culture model. J. Nutr. Biochem..

[26] Kamiloglu S., Capanoglu E., Grootaert C., Van Camp J. (2015). Anthocyanin Absorption and Metabolism by Human Intestinal Caco-2 Cells—A Review. Int. J. Mol. Sci..

[27] Fairweather-Tait S., Lynch S., Hotz C., Hurrell R.F., Abrahamse L., Beebe S., Bering S., Bukhave K., Glahn R., Hambidge M. (2005). The usefulness of in vitro models to predict the bioavailability of iron and zinc: A consensus statement from the HarvestPlus expert consultation. Int. J. Vitam. Nutr. Res..

[28] Tako E., Bar H., Glahn R. (2016). The combined application of the Caco-2 cell bioassay coupled with in vivo (*Gallus gallus*) feeding trial represents an effective approach to predicting Fe bioavailability in humans. Nutrients.

[29] Association of Official Analytical Chemists (AOAC) (2005). Official Methods of Analysis of AOAC International.

[30] Zand N., Chowdhry B.Z., Wray D.S., Pullen F.S., Snowden M.J. (2012). Elemental content of commercial ‘ready to-feed’ poultry and fish based infant foods in the UK. Food Chem..

[31] Bechoff A., Westby A., Owori C., Menya G., Dhuique-Mayer C., Dufour D., Tomlins K. (2010). Effect of drying and storage on the degradation of total carotenoids in orange-fleshed sweetpotato cultivars. J. Sci. Food Agric..

[32] Lee H.S., Coates G.A. (1987). Liquid chromatographic determination of vitamin C in commercial Florida citrus juices. J. Micronutr. Anal..

[33] Isabelle M., Lee B.L., Lim M.T., Koh W.-P., Huang D., Ong C.N. (2010). Antioxidant activity and profiles of common vegetables in Singapore. Food Chem..

[34] Christides T., Amagloh F.K., Coad J. (2015). Iron bioavailability and provitamin A from sweet potato- and cereal-based complementary foods. Foods.

[35] Glahn R.P., Lee O.A., Yeung A., Goldman M.I., Miller D.D. (1998). Caco-2 cell ferritin formation predicts nonradiolabeled food iron availability in an in vitro digestion Caco-2 cell culture model. J. Nutr..

[36] Yun S.M., Habicht J.P., Miller D.D., Glahn R.P. (2004). An in vitro digestion/Caco-2 cell culture system accurately predicts the effects of ascorbic acid and polyphenolic compounds on iron bioavailability in humans. J. Nutr..

[37] Sharp P., Tandy S., Yamaji S., Tennant J., Williams M., Singh Srai S.K. (2002). Rapid regulation of divalent metal transporter (DMT1) protein but not mRNA expression by non-haem iron in human intestinal Caco-2 cells. FEBS Lett..

[38] Sun H., Mu T., Xi L., Zhang M., Chen J. (2014). Sweet potato (*Ipomoea batatas* L.) leaves as nutritional and functional foods. Food Chem..

[39] Islam S.N., Nusrat T., Begum P., Ahsan M. (2016). Carotenoids and β-Carotene in orange fleshed sweet Potato: A possible solution to vitamin A deficiency. Food Chem..

[40] Van Jaarsveld P.J., Faber M., Tanumihardjo S.A., Nestel P., Lombard C.J., Benade A.J.S. (2005). β–carotene-rich orange-fleshed sweet potato improves the vitamin A status of primary school children assessed with the modified-relative-dose-response test. Am. J. Clin. Nutr..

[41] Tumwegamire S., Mwanga R.O.M., Andrade M., Low J.W., Kapinga R.E., Ssemakula G.N., Laurie S.M., Chipungu F.P., Ndirigue J., Agili S. (2014). Orange-Fleshed Sweetpotato for Africa: Catalogue 2014.

[42] Lartey A., Manu A., Brown K.H., Peerson J.M., Dewey K.G. (1999). A randomized, community-based trial of the effects of improved, centrally processed complementary foods on growth and micronutrient status of Ghanaian infants from 6 to 12 mo of age. Am. J. Clin. Nutr..

[43] Lartey A., Manu A., Brown K.H., Peerson J.M., Dewey K.G. (2000). Predictors of growth from 1 to 18 months among breast-fed Ghanaian infants. Eur. J. Clin. Nutr..

[44] Teucher B., Olivares M., Cori H. (2004). Enhancers of iron absorption: Ascorbic acid and other organic acids. Int. J. Vitam. Nutr. Res..

[45] Hallberg L., Brune M., Erlandsson M., Sandberg A.-S., Rossander-Hulten L. (1991). Calcium: Effect on different amounts on non heme-iron and heme-iron absorption in humans. Am. J. Clin. Nutr..

[46] Amagloh F.K., Brough L., Weber J.L., Mutukumira A.N., Hardacre A., Coad J. (2012). Sweetpotato-based complementary food would be less inhibitory on mineral absorption than a maize-based infant food assessed by compositional analysis. Int. J. Food Sci. Nutr..

[47] Amagloh F.K., Coad J. (2014). Orange-fleshed sweet potato-based infant food is a better source of dietary vitamin A than a maize-legume blend as complementary food. Food Nutr. Bull..

